# Editorial for the Special Issue: “Antimicrobial Resistance and Molecular Tracing of Foodborne Pathogens”

**DOI:** 10.3390/microorganisms10020390

**Published:** 2022-02-08

**Authors:** Jens André Hammerl

**Affiliations:** Department Biological Safety, German Federal Institute for Risk Assessment, Max-Dohrn Str. 8–10, 10589 Berlin, Germany; jens-andre.hammerl@bfr.bund.de; Tel.: +49-30-18412-24501

Foodborne pathogens are a major cause of diarrheal disease worldwide, but also constitute a severe threat for the spread of antimicrobial-resistant bacteria from livestock via food products to humans [1]. Due to an improper use of antibiotics in veterinary medicine, commensal, opportunistic and pathogenic bacteria were reported to successively arm up their genomes with antimicrobial/biocide resistance to adapt to the prevailing conditions in their respective environments. Reliable information about their occurrence, dynamics, adaption mechanisms and diversity is necessary and might lower their transmission to humans due to the use of adapted management strategies [2,3]. Within this Special Issue, an overview on some resistance-associated foodborne pathogens/indicator bacteria is given.

Despite various intervention measures in veterinary/human medicine, the development of antimicrobial resistances (AMR) is still on the rise. Particularly, bacteria of the ESKAPE (*Enterococcus* spp., *Staphylococcus aureus*, *Klebsiella pneumoniae*, *Acinetobacter baumannii*, *Pseudomonas aeruginosa*, *Enterobacter* spp.) group as well as *Escherichia coli* represent a major threat for human health based on their potential to exchange AMR with other bacteria [4]. This Special Issue addresses the impact of different Gram-negative/-positive bacteria and specific resistances associated with them. Among the available antibiotics, especially substances of the classes beta-lactams (i.e., cephalosporins, carbapenems), colistin, fluoroquinolones/quinolones, etc., exhibit a high impact for the treatment of Gram-negative bacteria associated with human infections [5,6]. However, some of these substances are not only restricted to human therapy but are also used to treat infections in livestock. So, the transmission of resistant bacteria from animals (livestock/pets) to other compartments is feasible. 

In several studies of this Special Issue, livestock was investigated for its relevance as a reservoir for antimicrobial resistances. In healthy broiler chicken from Korea, >90% of the investigated *E. coli* exhibited resistances against various antimicrobials [7]. For the majority of the extended-spectrum beta-lactamase (ESBL) or plasmid-mediated AmpC beta-lactamase (pAmpC)-producing *E. coli*, transmissible beta-lactam resistances were observed, which are associated with ISECP, IS903 and orf477 insertion sequences and IncI1, IncHI2, and/or IncFII plasmids. Interestingly, ESBL/pAmpC-producing *E. coli* often also carry plasmids encoding quinolone- or colistin-resistance genes. In another study on commensal and ESBL-producing *E. coli* from the German monitoring on zoonoses from livestock and food, the occurrence of *qnr* genes in fluoroquinolone-/quinolone-resistant *E. coli* was determined. Similar to the Korean *E. coli*, Juraschek et al. [8] also found a high number of fluoroquinolone-/quinolone-resistant isolates among ESBL-producing *E. coli* from livestock and food origin. So, the occurrence of *E. coli* co-carrying genes for both antibiotic classes seem to emerge. Furthermore, *qnr* was found in association with genes conferring decreased susceptibilities against quaternary ammonium compounds, which further arm them up against some disinfection products. Another interesting paper about clinically relevant *E. coli* from water samples of German poultry and pig slaughterhouses was provided by Savin et al. [9]. In addition to high numbers of multidrug-resistant *E. coli* (incl. resistances against 3rd/4th generation cephalosporins, fluoroquinolones), the authors also found clinically important isolates belonging to high-risk clones involved in human infections worldwide. Among them, ExPEC and UPEC pathotypes were detected, which might be transmitted to humans via food by inappropriate hygiene management during animal slaughtering. However, there is also a potential for an uncontrolled release to the environment via insufficiently treated wastewater.

In addition to the above-mentioned antibiotics, colistin also represents an important substance for human and animal therapy. Due to improper use (i.e., growth promoter of food animals) in the past, livestock currently represents a common reservoir for colistin-resistant *Enterobacterales* carrying transmissible *mcr* genes or alteration in chromosomal sequences associated with a resistance against colistin [10]. Investigation on the occurrence of bacterial colistin-resistances is also addressed in the Special Issue by three groups. Kubelová et al. [11] provide detailed insights into the diversity and virulence factors of *mcr*-1-positive *E. coli* from retail raw meat of the Czech Republic. While the analyzed isolates exhibit a broad genetic diversity (different phylogroups and multilocus sequence types), the isolates were assigned to the pathotypes APEC, ExPEC and UPEC. Regarding AMR spread, plasmidic-encoded *mcr* genes are of especially high importance. The impact of transmissible plasmids for the dissemination of *mcr* genes has been widely reported. In the study of Mechesso et al. [12], the authors focused on the dissection of *mcr*-3-carrying *E. coli* from diseased pigs from South Korea. There, different gene variants were identified (*mcr*-3.1/*mcr*-3.5), mainly carrying plasmids of the IncHI2 and IncP group, which were shown to be transmissible by conjugation for half of the analyzed isolates. In addition to *E. coli*, *Salmonella* is also recognized as a common host for colistin-resistant plasmids and a driver for their spread. Data on *Salmonella* were provided by Moon et al. [13] for livestock isolates collected between 2010 and 2018. Interestingly, the serovars *S*. Enteritidis and *S*. Gallinarum were found to be especially colistin-resistant. An *mcr*-1 carrying *S*. Typhimurium was described in detail. *mcr*-1 was shown to be encoded on an IncI2 plasmid, which could be successfully transmitted to *E*. *coli* by mating experiments.

The impact of biocides, used as disinfectants in animal husbandry, for supporting the selection and persistence of antimicrobial-resistant bacteria has been studied by Roedel et al. [14] on *E. coli* from broiler fattening farms. Despite the phenotypically and genotypically detected antimicrobial resistances and increased tolerance against biocides, no interlinkage was detected. However, in the analyzed genomes, various biocide and metal-resistance genes were detected on mobile genetic elements co-carrying antimicrobial resistances. Thus, transmission of both biocide and antimicrobial resistances via horizontal gene transfer is probable and might further force the persistence of multidrug-resistant isolates in animal husbandries via co-selection.

In the last decade, bacterial typing shifts from phenotypic testing to WGS and in silico analysis. Especially in antimicrobial resistance monitoring, the use of sequence-based technologies is on the rise for providing broader information on determinants associated with antimicrobial resistances and/or for performing retrospective in silico analyses on the prevailing datasets. However, actually the interpretation of the WGS datasets is challenging as a direct correlation of plasmid-associated AMR is often only possible using long-read sequencing approaches as shown by Juraschek et al. [15]. While all AMR genes could be reliably predicted, the different sequencing technologies used for the dissection of a selection of five *E. coli* in this study result in different genome sizes and plasmid contents, highlighting the necessity for further optimization and harmonization of the methodology and interpretation.

Bogaerts et al. [16] reported on the fluoroquinolone resistance in *Shigella* spp. from Belgium. The WGS analysis of a broad spectrum of isolates collected between 2013 and 2018 showed both an association of shigellosis infections to travels (esp. to Asia/Africa) and also reveals the presence of domestically circulating strains. This study also highlights the benefit of WGS data for typing and surveillance of this pathogen.

To monitor the dynamics of antimicrobial resistances in livestock, many groups use commensal bacteria, such as *E. coli* or enterococci, as reliable indicators. From South Korea, antimicrobial resistance profiles and resistance trends of *Enterococcus faecium* and *E. faecalis* collected between 2010 and 2019 were described by Kim et al. [17]. Overall, isolates of both species, from pig and chicken, exhibited high rates of antimicrobial resistances against some substances. Due to the increasing trends, which were found for some antibiotics, the potential risk to public health posed by enterococci from food animals is steadily increasing. In another study, the occurrence of different enterococci and their antimicrobial resistance content was determined for South Africa by Badul et al. [18]. Among the determined species (i.e., *E. faecalis*, *E. faecium*, *E. casseliflavus*, *E. gallinarum*, and other *Enterococcus* sp.), high numbers exhibit multiple resistances, but lack resistance against vancomycin, teicoplanin, tigecycline and linezolid. Based on their results, the authors concluded that intensive pig farming will develop a reservoir of antibiotic-resistant bacteria that might be further spread to the working personnel by direct contact to colonized animals or to consumers via the ingestion of contaminated food. Thus, suitable guidelines for antimicrobial use and management strategies for controlling hygiene is mandatory. Further insights into phenicol–oxazolidinone resistances in linezolid-resistant enterococci from food-producing animals were also provided for South Korea. Na et al. [19] provided specific data on the occurrence of *poxtA* on a broad range of *E. faecium*/*faecalis* collected in South Korea between 2008 and 2018. *poxtA*-positive enterococci were mainly detected in chickens. However, as the gene was shown to be transmissible, a further spread might also affect other food animals or finally humans in the future.

By Cao et al. [20], a novel real-time *Clostridium perfringens* α toxin (CPA) detection methodology was reported, based on a combination of nano-silica microspheres combined with smartphone image processing technology. The method includes silica microspheres coupled with the CPA_C3_ antibodies catching accessible toxins, which was incubated with the fluorescent-labeled antibody of CPA_N_ and further visualized in a cell phone app. By using this assay, toxin concentrations of >32.8 ng/mL can be detected after 90 min. The authors recommended the method for food investigations.

Another interesting study provides an overview about the potential of intra-farm transmission of livestock-associated MRSA sequence type ST398 isolates on German dairy farms by Lienen et al. [21]. In a One Health approach, isolates from different sectors were subjected to WGS and bioinformatics analysis. The phylogenic allocation of the isolates confirmed farm-specific transmissions, which not only can affect milk used for the feeding of calves, but also the environment or surroundings of the stable/farm and other animals, as well as the farm personnel. To avoid a spread of germs posing a health threat, the necessity for strict hygiene in stables and farms as well as suitable management strategies were requested.

One of the most important foodborne pathogens is *Campylobacter* spp. As well as its potential for gastrointestinal infections in human, thermophilic *Campylobacter* are also studied regarding their antimicrobial resistance patterns. In the study of Choi et al. [22] in Korea, the majority of *C. coli* isolates from fecal and carcass samples of pigs and chickens collected between 2010 and 2018 exhibit high levels of MDR (incl. resistance against ciprofloxacin, nalidixic acid, and tetracycline). Resistance development in *Campylobacter* might provide a selection benefit for the survival of the pathogens in livestock. Thus, a prudent use of antimicrobials will contribute to a reduction in the health risk for human *C. coli* infections.

In order to address the impact of antimicrobial resistances for human health, this Special Issue also benefits from human surveillance data provided by Brandl et al. [23]. Incidence rates for invasive infections with MRSA, for infection or colonizations with carbapenem-non-susceptible *Acinetobacter* spp. and Enterobacterales, indicated that men in Germany show double the risk of infection and colonization with AMR pathogens than women. This result will help to develop gender-stratified approaches for a better detection or infection prevention.

## References

[1] Aarestrup F.M. (2015). The livestock reservoir for antimicrobial resistance: A personal view on changing patterns of risks, effects of interventions and the way forward. Philos. Trans. R. Soc. B: Biol. Sci..

[2] EFSA (2020). The European Union summary report on antimicrobial resistance in zoonotic and indicator bacteria from humans, animals and food in 2017/2018. EFSA J..

[3] WHO (2014). Antimicrobial Resistance Global Reporton Surveillance.

[4] Savin M., Bierbaum G., Hammerl J.A., Heinemann C., Parcina M., Sib E., Voigt A., Kreyenschmidt J. (2020). Antibiotic-resistant bacteria and antimicrobial residues in wastewater and process water from German pig slaughterhouses and their receiving municipal wastewater treatment plants. Sci. Total Environ..

[5] Carattoli A. (2008). Animal reservoirs for extended spectrum beta-lactamase producers. Clin. Microbiol. Infect..

[6] Robicsek A., A Jacoby G., Hooper D.C. (2006). The worldwide emergence of plasmid-mediated quinolone resistance. Lancet Infect. Dis..

[7] Song H.-J., Moon D.C., Mechesso A.F., Kang H.Y., Kim M.H., Choi J.-H., Kim S.-J., Yoon S.-S., Lim S.-K. (2020). Resistance Profiling and Molecular Characterization of Extended-Spectrum/Plasmid-Mediated AmpC β-Lactamase-Producing *Escherichia coli* Isolated from Healthy Broiler Chickens in South Korea. Microorganisms.

[8] Juraschek K., Deneke C., Schmoger S., Grobbel M., Malorny B., Käsbohrer A., Schwarz S., Meemken D., Hammerl J.A. (2017). Phenotypic and Genotypic Properties of Fluoroquinolone-Resistant, qnr-Carrying Escherichia coli Isolated from the German Food Chain in 2017. Microorganisms.

[9] Savin M., Bierbaum G., Kreyenschmidt J., Schmithausen R., Sib E., Schmoger S., Käsbohrer A., Hammerl J. (2021). Clinically Relevant *Escherichia coli* Isolates from Process Waters and Wastewater of Poultry and Pig Slaughterhouses in Germany. Microorganisms.

[10] Binsker U., Käsbohrer A., A Hammerl J. (2021). Global colistin use: A review of the emergence of resistant Enterobacterales and the impact on their genetic basis. FEMS Microbiol. Rev..

[11] Kubelová M., Koláčková I., Gelbíčová T., Florianová M., Kalová A., Karpíšková R. (2021). Virulence Properties of *mcr*-1-Positive *Escherichia coli* Isolated from Retail Poultry Meat. Microorganisms.

[12] Mechesso A.F., Moon D.C., Kang H.Y., Song H.-J., Kim S.-J., Choi J.-H., Kim M.H., Na S.H., Kim H.-Y., Jung B.Y. (2020). Emergence of *mcr-3 carrying Escherichia coli* in Diseased Pigs in South Korea. Microorganisms.

[13] Moon D., Kim S.-J., Mechesso A., Kang H., Song H.-J., Choi J.-H., Yoon S.-S., Lim S.-K. (2021). Mobile Colistin Resistance Gene *mcr*-*1* Detected on an IncI2 Plasmid in *Salmonella* Typhimurium Sequence Type 19 from a Healthy Pig in South Korea. Microorganisms.

[14] Roedel A., Vincze S., Projahn M., Roesler U., Robé C., Hammerl J.A., Noll M., Al Dahouk S., Dieckmann R. (2021). Genetic but No Phenotypic Associations between Biocide Tolerance and Antibiotic Resistance in *Escherichia coli* from German Broiler Fattening Farms. Microorganisms.

[15] Juraschek K., Borowiak M., Tausch S.H., Malorny B., Käsbohrer A., Otani S., Schwarz S., Meemken D., Deneke C., Hammeri J.A. (2021). Outcome of different sequencing and assembly approaches on the detection of plasmids and localization of antimicrobial resistance genes in commensal Escherichia coli. Microorganisms.

[16] Bogaerts B., Winand R., Van Braekel J., Mattheus W., De Keersmaecker S., Roosens N., Marchal K., Vanneste K., Ceyssens P.-J. (2021). Phylogenomic Investigation of Increasing Fluoroquinolone Resistance among Belgian Cases of Shigellosis between 2013 and 2018 Indicates Both Travel-Related Imports and Domestic Circulation. Microorganisms.

[17] Kim M., Moon D., Kim S.-J., Mechesso A., Song H.-J., Kang H., Choi J.-H., Yoon S.-S., Lim S.-K. (2021). Nationwide Surveillance on Antimicrobial Resistance Profiles of *Enterococcus faecium* and *Enterococcus faecalis* Isolated from Healthy Food Animals in South Korea, 2010 to 2019. Microorganisms.

[18] Badul S., Abia A.L.K., Amoako D.G., Perrett K., Bester L.A., Essack S.Y. (2021). From the farms to the dining table: The distribution and molecular characteristics of antibiotic-resistant Enterococcus spp. in intensive pig farming in South Africa. Microorganisms.

[19] Na S.-H., Moon D.-C., Kim M.-H., Kang H.-Y., Kim S.-J., Choi J.-H., Mechesso A.-F., Yoon S.-S., Lim S.-K. (2020). Detection of the Phenicol–Oxazolidinone Resistance Gene *poxtA* in *Enterococcus faecium* and *Enterococcus faecalis* from Food-Producing Animals during 2008–2018 in Korea. Microorganisms.

[20] Cao A., Chi H., Shi J., Sun R., Du K., Song Y., Zhu M., Zhang L., Huang J. (2020). Visual Detection of *Clostridium perfringens* Alpha Toxin by Combining Nanometer Microspheres with Smart Phones. Microorganisms.

[21] Lienen T., Schnitt A., Cuny C., Maurischat S., Tenhagen B.-A. (2021). Phylogenetic Tracking of LA-MRSA ST398 Intra-Farm Transmission among Animals, Humans and the Environment on German Dairy Farms. Microorganisms.

[22] Choi J.-H., Moon D.C., Mechesso A.F., Kang H.Y., Kim S.-J., Song H.-J., Yoon S.-S., Lim S.-K. (2021). Antimicrobial Resistance Profiles and Macrolide Resistance Mechanisms of *Campylobacter coli* Isolated from Pigs and Chickens. Microorganisms.

[23] Brandl M., Hoffmann A., Willrich N., Reuss A., Reichert F., Walter J., Eckmanns T., Haller S. (2021). Bugs That Can Resist Antibiotics but Not Men: Gender-Specific Differences in Notified Infections and Colonisations in Germany, 2010–2019. Microorganisms.

